# Asymmetric dimethylarginine induces maladaptive function of the blood-brain barrier

**DOI:** 10.3389/fcell.2024.1476386

**Published:** 2024-10-09

**Authors:** Tetyana P. Buzhdygan, Servio H. Ramirez, Miroslav N. Nenov

**Affiliations:** ^1^ Department of Neural Sciences, Alzheimer’s Center at Temple, Temple University Lewis Katz School of Medicine, Philadelphia, PA, United States; ^2^ Department of Pathology, Immunology and Laboratory Medicine, College of Medicine, University of Florida, Gainesville, FL, United States; ^3^ Department of Psychology and Neuroscience, College of Liberal Arts, Temple University, Philadelphia, PA, United States

**Keywords:** human BBB model, monocyte adhesion and migration, asymmetric dimethylarginine, ADMA, cytokines, VCAM-1, RANTES, VEGF-A

## Abstract

Growing body of evidence suggests that cardiovascular risk factor, asymmetric dimethylarginine (ADMA), can be implicated in the pathogenesis of neurodegenerative and psychiatric disorders. In part, ADMA can affect brain health negatively modulating critical functions of the blood-brain barrier (BBB). The precise mechanisms and consequences of ADMA action on the cerebral vasculature remains unexplored. Here, we evaluated ADMA-induced maladaptation of BBB functions by analyzing real time electrical cell-substrate impedance, paracellular permeability, immune-endothelial interactions, and inflammatory cytokines production by primary human brain microvascular endothelial cells (hBMVEC) treated with ADMA. We found that ADMA disrupted physical barrier function as evident by significant decrease in electrical resistance and increase in paracellular permeability of hBMVEC monolayers. Next, ADMA triggered immune-endothelial interactions since adhesion of primary human monocytes and their extravasation across the endothelial monolayer both were significantly elevated upon treatment with ADMA. Increased levels of cell adhesion molecules (VCAM-1 and RANTES), VEGF-A and inflammatory cytokines (IL-1β, TNF-α, IL-6, IL-10, IL-4, IL-2, IL-13, IL-12p70) characterize ADMA-induced hBMVEC dysfunction as inflammatory. Overall, our data suggest that ADMA can impair BBB functions disrupting the endothelial barrier and eliciting neuroinflammatory and neuroimmune responses.

## Introduction

Asymmetric dimethylarginine (ADMA), which is an endogenous inhibitor of nitric oxide synthase (NOS), is one of the cardiovascular risk factors that induce endothelial dysfunction in various pathophysiological conditions (Böger, 2005; Dorszewska, 2014). Elevation in plasma ADMA concentration is also associated with a risk of development of neurodegenerative and psychiatric disorders, the finding that can be attributed to negative effects of ADMA on the brain microvasculature (Braun et al., 2021; Khan et al., 2021). It was found that cerebral blood vessels are highly sensitive to changes in ADMA concentration as asymmetric dimethylarginine effectively blocks acetylcholine-induced vasodilation of cerebral arterioles via inhibition of brain NOS (Faraci et al., 1995). Administration of exogenous ADMA significantly decreases cerebral blood flow in healthy men, while changes in ADMA/arginine ratio are indicative of microangiopathy-related cerebral damage in elders (Kielstein et al., 2006; Notsu et al., 2009). High ADMA level is correlated with the cognitive decline in patients with cerebral small vessel disease lesion (Gao et al., 2015). In addition, it was recently found that elevation in ADMA level compromises blood-brain barrier (BBB) integrity and affects BBB function in animal models of neurodegeneration and multiple sclerosis (Choi et al., 2020; Singh et al., 2021).

ADMA is produced by protein-arginine methyltransferases from proteins methylated at arginine residues. In cells ADMA is metabolized by special group of enzymes–dimethylarginine dimethylaminohydrolase (DDAH) type-1 and type-2 that are presented in both brain endothelial and neuronal cell types (Cooke and Ghebremariam, 2011; Kozlova et al., 2022). The pathological effect of ADMA on brain vasculature can be associated with either an increase in ADMA concentration and/or a decrease in DDAH function/expression levels. Thus, it was found that in TgSwDl mouse, an animal model of Alzheimer’s disease, serum ADMA concentration progressively increased in an age-dependent manner. This elevation was accompanied by decreased levels in DDAH-2 in TgSwDl mice. Furthermore, TgSwDl mice treated with exogenous ADMA showed further progression of neurovascular pathology as evidenced by enhanced blood-brain barrier leakage, reduction in BBB integrity, and decreased levels of tight junction proteins such as ZO-1 and Claudin-1 (Choi et al., 2020). Another study showed that ischemic stress induced by middle cerebral artery occlusion-reperfusion significantly potentiated BBB disruption, elevated BBB leakage, and decreased tight junction protein levels in DDAH-1 knock-out mice (Yichen et al., 2021). It was recently found that continuous infusion with ADMA impairs spatial memory and decreases protein levels important for BBB integrity as Claudin-1, Occludin and ZO-1 in hippocampus of young male rats (Hsu et al., 2023). However, importance of ADMA in regulation of BBB function and cognitive performance in health and neurodegenerative diseases is primarily studied in animal models. There are few studies showing that treatment of human brain microvascular endothelial cells with 30–100 µM of ADMA for 24 h significantly affected endothelial cell viability due to activation of NF-kB signaling pathway, increase in reactive oxygen species production (ROS) and reduction in Bcl-2, resulting in activation of pro-apoptotic caspase-3 (Ma et al., 2015; Li et al., 2016).

Taking together these results indicate that ADMA plays an important role in the regulation of BBB function and that further studies are needed to shed light on ADMA action that underlies BBB dysfunction and impairment in brain health. Previous studies utilizing human BBB models were focused upon the mechanism of ADMA transport and signaling pathways underlying cytotoxic effects of ADMA at high concentrations (Ma et al., 2015; Li et al., 2016; Watson et al., 2016). In our study, we opted to use physiologically relevant concentration of ADMA to further characterize its pathophysiological action on barrier function, immune-endothelial interactions, and inflammatory status of the BBB. For added rigor and enhanced translatability of our research, we utilized primary human cells isolated from 5-6 donors (human brain microvascular endothelial cells (hBMVEC) and primary human monocytes) to account for inter-donor variability and biological differences.

## Materials and methods

### Primary hBMVEC

Primary human brain microvascular endothelial cells (hBMVEC) were isolated from fetal brain tissue as described earlier (Andrews et al., 2018). Fetal brain tissue was provided under informed consent by the Laboratory of Developmental Biology (University of Washington, Seattle, WA) with approval granted by Temple University (Philadelphia, PA) Institutional Review Board and in full compliance to the National Institutes of Health’s (NIH) ethical guidelines. Human fBMVEC were maintained in complete growth medium (EBM-2 supplemented with EGM-2MV SingleQuots, Lonza, Cat No CC-3156 and CC-4147) in rat-tail collagen I (BD Biosciences) flasks at 37°C, 5% CO2, and 100% humidity. For all experiments only low passages of cells (passage 3–7) were used. For barrier formation, confluent monolayers were deprived of growth factors and maintained in EBM2 supplemented with 5% FBS for three to 5 days before experiment. Serum-free, growth-factor free medium (EBM-2) was used to dilute drugs for cell treatment.

### Human primary monocytes

Primary human peripheral blood monocytes were obtained from HIV-1 and hepatitis B seronegative donors and provided by University of Nebraska Medical Center. The monocytes were maintained in DMEM media supplemented with 10% heat inactivated pooled fetal bovine serum, penicillin 100 (U/mL), streptomycin (100 U/mL) and L-glutamine (2 mM) and used within 24 h of isolation.

### Cell viability assay

ADMA cytotoxicity was evaluated using LIVE/DEAD viability/cytotoxicity assay (Life Technologies, L32250). Human primary monocytes were plated on 96-well plate at the density of 25,000 cells per well, allowed to attach overnight and treated with. hBMVEC were seeded on 96-well plate, allowed to form confluent monolayer, and then treated with 10 μM ADMA or 0.1% saponin for 24 h or 72 h. Live and dead cells were detected as per manufacturer protocol. Data was acquired at excitation and emission wavelengths of 495/515 nm (Calcein-AM, live cells indicator) and 660/682 nm (ethidium homodimer-1, dead cells indicator). Data is presented as ratio of live or dead cells in ADMA-treated wells normalized to negative (untreated cells) or positive (cells treated with saponin) controls.

### Electric cell-substrate impedance sensing (ECIS) assay

Real-time changes of transendothelial electrical resistance were monitored using the electric cell-substrate impedance sensing (ECIS) Z-Theta 96 Well Array Station (Applied Biophysics). ECIS was recorded using the multiple frequency/time (MFT) mode to continuously monitor changes in impedance over spectrum of frequencies (400 Hz–48,000 Hz). 96W20idf PET arrays were incubated with 10 mM cysteine solution to stabilize gold electrodes followed by coating with rat-tail collagen type 1. HBMVEC were plated at the density of 10,000 cells per each well with one well left cell-free for model purpose and grown until confluent monolayer and functional barrier were formed as indicated by stable resistance >600 Ω at 4,000 Hz and capacitance <20 mA at frequency 400 Hz. For the growing phase (2 days) cells were maintained in complete growth medium, for the barrier formation phase (4–5 days) cells were maintained in EBM2 supplemented with 5% FBS with 50% of medium changed every second day. After the functional monolayer was formed and stable baseline resistance was reached (>600 Ω), ADMA at various concentrations (1 μM, 10 μM, 100 μM) or 100 ng/mL TNFα were added to quadruplicated wells and the recording continued for 36 h. Intercellular barrier resistance component was extracted using the Rb (barrier resistance) modeling function of the ECIS software.

### Paracellular permeability assay

To evaluate the paracellular permeability, cells were seeded at the density of 10,000 cells per collagen I-coated Transwell insert (pore size 0.4 μm, diameter 0.33 cm^2^, Corning) in the 250 μL of complete growth medium. Basolateral chambers were filled with 500 μL of complete growth medium. After 2 days, medium was changed to EBM2 with 5% FBS to allow proper barrier formation (5 days) with 50% medium changed every 2 days. After monolayers were formed, hBMVEC were serum starved for 1 h and then incubated with 10 μM ADMA for 30 min. FITC-conjugated 4 kDa dextran (Sigma) was added to the apical chamber to the final concentration of 2 mg/mL, and 3 h later, medium from the basolateral chamber was carefully removed and fluorescence was measured at 525 nm using a SpectraMax M5e (Molecular Devices). Apparent permeability coefficient (Papp) was determined using the following equation: Papp=(ΔQ/Δt)/AC0, where (ΔQ/Δt) is the steady-state flux of tracer (mg/s), A is the surface area of the permeable insert (0.33 cm^2^), and C0 is the initial concentration in the donor chamber (in mg/mL) as described.

### Monocyte adhesion assay

HBMVEC were plated on collagen-coated 96-well plates at a density of 2.5 ×10^4^ cells/well. After formation of confluent monolayers, hBMVEC were treated with 10 μM ADMA or 100 ng/mL TNFα for 24 h. The endothelial monolayers were rinsed of all treatments and fluorescently labeled monocytes (10^5^ cells/well loaded with calcein-AM (Invitrogen) at 5 μM/1×10^6^ cells for 45 min) were applied and co-incubated for 40 min at 37°C. After adhesion, the monolayers were washed three times with PBS (with Ca^2+^ and Mg^2+^) and fluorescence was acquired on a fluorescence plate reader, Spectramax M5 (Molecular Devices, Sunnyvale, CA). The data was calculated based on the standard curve derived from fluorescent intensity of known amounts of labeled monocytes. Results are represented as number of cells adhered to the endothelial monolayers.

### Monocyte transendothelial extravasation assay

HBMVEC were plated on rat-tail collagen type I coated FluoroBlok™ HTS 96-well inserts with 8 μm pores (Corning) at a density of 4×10^3^ cells/insert. After formation of confluent monolayers, hBMVEC were treated with 10 μM ADMA or 100 ng/mL TNFα for 24 h. On the day of assay, the endothelial monolayers were rinsed of all treatments and relevant β-chemokine, recombinant human monocyte chemotactive protein (MCP) −1 (CCL2/MCP-1, 30 ng/mL, R&D) was added into the lower chamber to create a chemokine gradient present in neuroinflammatory disorders. Calcein-AM labeled monocytes (1×10^4^ cells/insert were added to the upper chamber, and migration was allowed to continue up to 2 h at 37°C. Fluorescence was acquired on a fluorescence plate reader, Spectramax M5 (Molecular Devices, Sunnyvale, CA). The data was calculated based on the standard curve derived from fluorescent intensity of known amounts of labeled monocytes. Results are represented as number of cells adhered to the endothelial monolayers.

### ELISA

To examine the concentration of growth factors secreted by hBMVEC, confluent monolayers were treated with 10 μM ADMA for 24 h. Cell culture supernatant was briefly centrifugated (5 min at 2000 g) to pellet cell debris and then analyzed using Human VEGF A Quantikine ELISA kit (RnD Systems, Cat No DVE00), Human CCL2/MCP-1 Quantikine ELISA kit (RnD Systems, Cat No DCP00), and Human CCL5/RANTES Quantikine ELISA kit (RnD Systems, Cat No DRN00B) as described in manufacturer’s protocols.

### MSD proinflammatory cytokine expression assay

To examine the concentration of proinflammatory cytokines secreted by hBMVEC, confluent monolayers were treated with 10 μM ADMA for 24 h. Cell culture supernatant was briefly centrifugated (5 min at 2000 g) to pellet cell debris and then analyzed using V-PLEX Proinflammatory Panel I Human kit (MesoScale Diagnostics Cat No K15049D) as described in the manufacturer’s protocol.

### Flow cytometry

HBMVECs plated in 12-well dishes were grown to confluency and treated with 10 µM of ADMA or 100 ng/mL of TNF-α for 24 h. After treatment, cells were washed with calcium and magnesium-free phosphate buffer saline (PBS) and detached with accutase for 1–2 min at 37°C. Cells were then pelleted by centrifugation at 1,000 rpm for 5 min and resuspended in the Fixation Buffer (eBioscience/Thermo Fisher) for 30 min. Following fixation, cells were washed with Flow Cytometry Buffer (5% FBS, 0.1% sodium azide) and pelleted again. Cells were resuspended for 30 min in 100 μL of Flow Cytometry Buffer, containing 5 μL of anti-ICAM-1 (BioLegend, Cat No 353116), 5 μL of PECAM (Biolegend, Cat No 303114), 5 μL of ALCAM (Biolegend, Cat no 343906) and 5 μL of anti-VCAM-1 (RnD Systems, BBA22) antibodies. Cells were then washed, pelleted, and resuspended in Flow Cytometry Buffer for FACS analysis. 10,000 events per sample were acquired with a FACS BD Canto II flow cytometer (BD Biosciences) and data was then analyzed with FlowJo software.

### Statistical analysis

**FIGURE 1:**
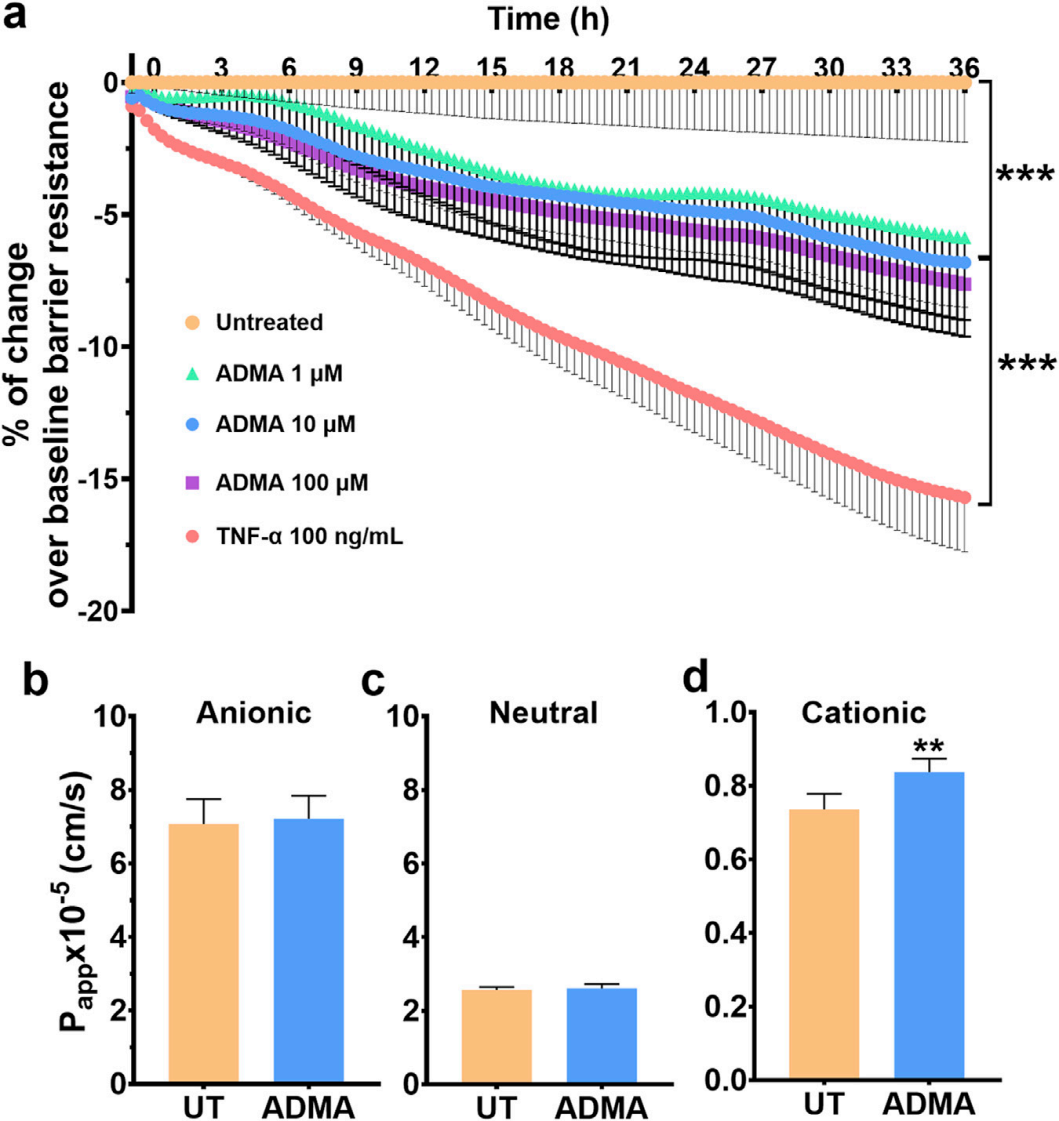
ADMA compromises barrier function of hBMVEC. **(A)**. Barrier electrical resistance was modelled based on continuous cell-substrate impedance readings recorded at six frequencies (400Hz–48 kHz) every 6 min for the duration of the time shown. Endothelial monolayers were treated with 1, 10 and 100 µM ADMA, or 100 ng/mL TNFα, or left untreated to serve as a baseline. Treatments were initiated at 0 timepoint. Each data point is represented as the percentage of the mean value ±SEM. Experiments were independently performed three times, primary cells from four different donors were used (n = 14). **(B–D)**. Barrier permeability of small molecular tracer was modelled using FITC-conjugated dextrans (4 kDa) of various charges: anionic **(B)**, neutral **(C)**, cationic **(D)**. Endothelial monolayers were treated with 10 μM ADMA or left untreated to serve as a baseline. Each data point is represented as the apparent permeability coefficient (Papp (x10^−5^), mean value ± SEM). Primary cells from five different donors were used (n = 20).

**FIGURE 2:**
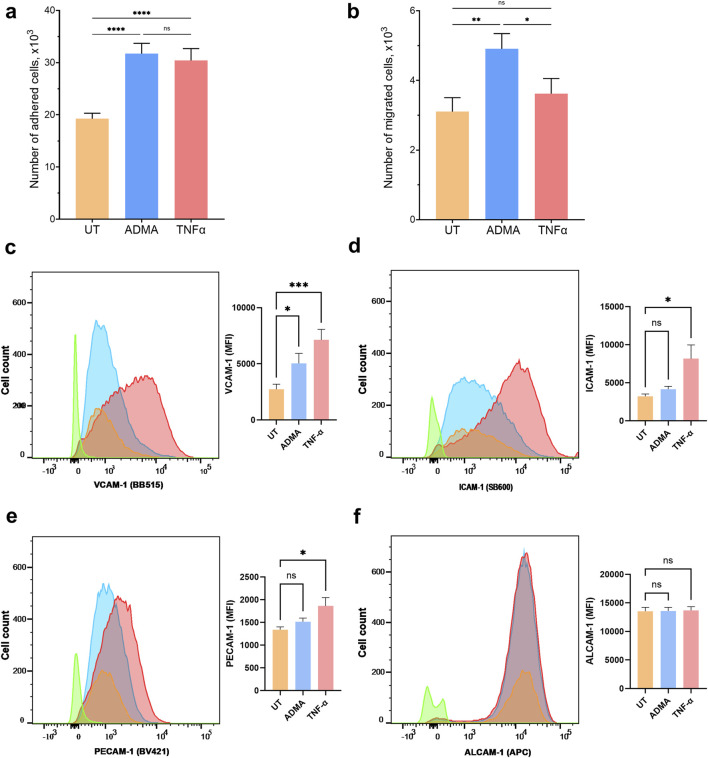
ADMA increases monocyte adhesion and migration across the BBB and enhances cell surface expression of cell adhesion molecule VCAM-1 in hBMVEC. Primary human monocytes adhesion **(A)** and migration **(B)** across the hBMVEC monolayers. Results are presented as number of adhered or migrated cells (x1,000, mean ± SEM). Experiments were performed using hBMVEC isolated from five different donors and human monocytes from two different donors. For adhesion experiment **(A)** n = 32, and for migration experiment **(B)** n = 12. FACS flow cytometry was used to measure the expression of extracellular cell adhesion molecules on hBMVEC treated with 10 µM ADMA or 100 ng/mL TNFα for 24 h **(C)** Representative histogram and bar graph quantification of mean fluorescent intensity (MFI) for VCAM-1 surface expression. **(D)** Representative histogram and bar graph quantification of MFI for ICAM-1 surface expression. **(E)** Representative histogram and bar graph quantification of MFI for PECAM-1 surface expression. **(F)** Representative histogram and bar graph quantification of MFI for ALCAM-1 surface expression. Data shown as the mean number of positive cells ±SEM. Experiments were performed using primary cells from four different donors (n = 8).

**FIGURE 3:**
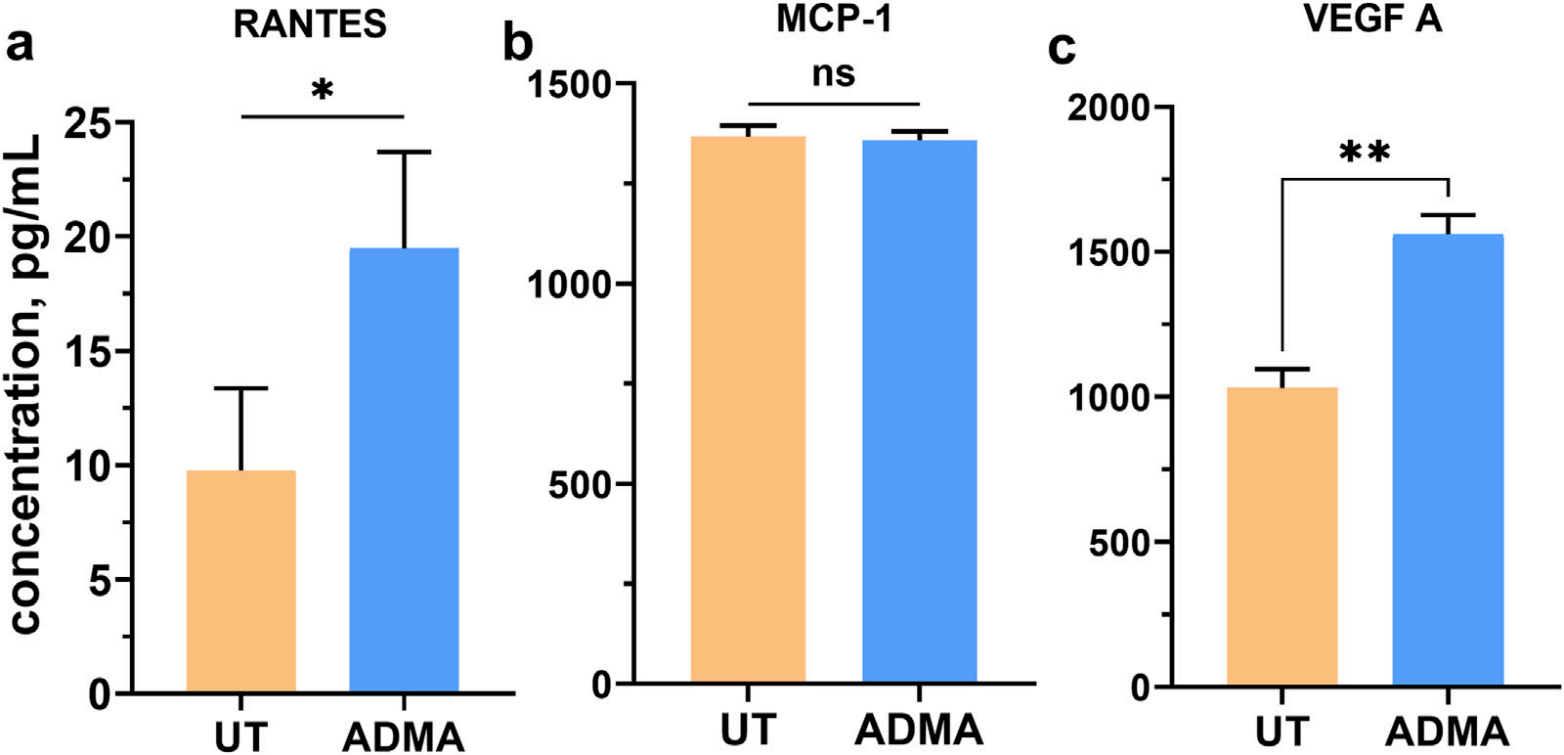
ADMA induces expression of angiogenic factors in hBMVEC. **(A)** ELISA was used to measure the concentration of secreted RANTES **(A)**, MCP-1 **(B)** and angiogenic factor VEGFA **(C)** in cell culture medium of hBMVEC treated with 10 µM ADMA for 24 h. Data shown as the mean concentration ±SEM expressed in pg/mL of cell supernatant. Experiments were performed using primary hBMVEC from four different donors (n = 8).

The experiments were independently performed multiple times (at least three times for all the data shown) to allow statistical analyses. Within each individual experimental set, primary hBMVEC from at least three donors were used. All data represent matched expriments (treatment group was matched to control group for hBMVEC isolated from the same donor). Paired t-test or Wilcoxon test (for data that did not pass normal distribution test) were used to analyze two groups (Figures 1B–D; Figures 2, 3). Repeated measures (RM) one-way ANOVA followed by *post hoc* Tukey’s or Fisher’s LSD test or Restricted Maximum Likelihood (REML) mixed-effects model followed by *post hoc* Dunnett’s test were used when multiple group comparisons were performed against a reference control (Figures 1A, 2; Supplementary Figure S2). Two-way ANOVA with *post hoc* Tukey’s test was used for viability assay in Supplementary Figure S1. Results are expressed as mean ± SEM with differences considered significant at p < 0.05. The data collected was analyzed using Prism v10.0 (GraphPad Software, San Diego, CA).

## Results

### ADMA does not induce cellular toxicity in hBMVEC

Previous studies showed that ADMA causes cytotoxicity and programmed cell death in hBMVEC cultures at high concentrations up to 100 µM (Ma et al., 2015; Li et al., 2016). For our experiments we seek to study the effects of 10 μM ADMA as it is reflective of intracellular concentration of this molecule at physiological conditions in endothelial cells and a brain tissue (Böger et al., 2000; Cardounel and Zweier, 2002; Teerlink et al., 2009). We assessed the effect of 10 μM ADMA on hBMVEC viability at 24 h and 72 h timepoints using Calcein-AM Live/Dead Cytotoxicity assay (Promega) and saponin as a positive control for dead cells. Our results showed no evidence of cytotoxicity with 10 μM ADMA at timepoints tested. After 24 h, the percent of viable cells in untreated group was 100% ± 3.990%, treated with saponin 0.008% ± 0.118% (p < 0.001), 10 μM ADMA 100.2% ± 6.721% (p > 0.05 with two-way ANOVA followed by Tukey’s post-hoc test; Supplementary Figure S1A) and after 72 h treatment the percent of viable cells in untreated group was 100% ± 3.732%, treated with saponin 0.008% ± 0.117% (p < 0.001), 10 μM ADMA 96.510% ± 8.008% (p > 0.05 with two-way ANOVA followed by Tukey’s post-hoc test; Supplementary Figure S1B). Overall, our results indicate that ADMA does not impact the viability of primary hBMVEC.

### ADMA impairs barrier properties of the hBMVEC

Next, we seek to examine the effect of ADMA on the barrier properties of the human BBB *in vitro*. To this end we evaluated electrical resistance (Rb) and paracellular permeability (PP) of endothelial monolayers. For Rb measurements, hBMVECs were plated on 96 well array with each well containing 20 interdigitated gold electrodes (Applied BioPhysics) that allow continuous measurement of electrical resistance from ∼8,000 cells. The baseline resistance measured at 4,000 Hz was at least 600 Ω for each donor. As shown in Figure 1A, hBMVEC monolayers treated with 1, 10 or 100 μM ADMA for 36 h sustained significant loss of electrical resistance. For instance, the area under the curve (AUC) for 10 μM of ADMA was 171.4 ± 13.8, p < 0.0001 with RM one-way ANOVA followed by Tukey’s *post hoc* test) and TNFα (AUC 385.9 ± 13.3, p < 0.0001 with RM one-way ANOVA followed by Tukey’s *post hoc* test). Then we studied the dose-response of ADMA action at individual time points. We found that all three tested concentrations showed a similar level of efficacy over the time course of 36 h. The difference was mainly in the time-dependency of observed effects (Supplementary Figure S2). Thus, at 1 h time point 1 μM ADMA did not show significant difference compared to baseline control (−0.55% ± 0.33%, p > 0.05 with Mixed-effects model followed by Dunnett’s *post hoc* test; Supplementary Figure S1A) while 10 and 100 μM concentrations both showed significant reduction in electrical resistance compared to baseline control (−1.088% ± 0.2666% for 10 μM ADMA and −1.032 ± 0.3016 for 100 μM ADMA, p < 0.01 with Mixed-effects model followed by Dunnett’s *post hoc* test; Supplementary Figure S1A). The negative effect of all tested concentrations continues to grow slowly and with the same pattern. At 9 h time point the effect of 1 μM ADMA was still insignificant (−1.626% ± 1.484%, p > 0.05 with Mixed-effects model followed by Dunnett’s *post hoc* test; Supplementary Figure S1C) and 10 and 100 μM ADMA exert more negative significant effect −2.808% ± 0.9649% for 10 μM ADMA and −3.260% ± 1.084% for 100 μM ADMA, p< 0.05 and p < 0.01 with Mixed-effects model followed by Dunnett’s *post hoc* test; Supplementary Figure S1C). The negative effect of ADMA continued to grow and reach significance for all three tested concentrations at 18 h time point (−3.955% ± 2.150% for 1 μM ADMA, −4.282% ± 1.429% for 10 μM ADMA and −4.900% ± 1.593% for 100 μM ADMA p < 0.05 and p < 0.01 with Mixed-effects model followed by Dunnett’s *post hoc* test; Supplementary Figure S1D). Finaly, ADMA effect reached its maximum level for all concentrations at 36 h time point (−5.880% ± 3.108% for 1 μM ADMA, −6.820% ± 1.683% for 10 μM ADMA and −7.637% ± 1.982% for 100 μM ADMA p < 0.05 and p < 0.01 with Mixed-effects model followed by Dunnett’s *post hoc* test; Supplementary Figure S1F).

To determine whether ADMA alters molecular flux across tight junctions, we performed a paracellular permeability assay with 4 kDa FITC-conjugated dextrans. Treatment with ADMA resulted in a significant increase of paracellular permeability 3 h after the application for cationic (positively charged) tracer only (Papp (x10^−5^) for untreated 0.734 x ± 0.043; for ADMA-treated 0.837 ± 0.037, p = 0.0017 with paired t-test, while the permeability for neutral and anionic tracers remained unchanged (for neutral dextran Papp (x10^−5^) for untreated 2.563 ± 0.0797 and for ADMA-treated 2.610 ± 0.115 (p > 0.05 with paired t-test); for anionic dextran Papp (x10^−5^) for untreated 7.072 ± 0.679 and for ADMA-treated 7.217 ± 0.625 (p > 0.05 with paired t-test). Figure 1B–D show paracellular permeability of anionic, neutral, and cationic FITC-dextran respectively. These results suggest that ADMA can cause an increase in the permeability of cerebral endothelial barriers to small cationic molecules what can underlay subsequent reduction in barrier electrical resistance.

### ADMA increases monocytes adhesion and extravasation across the BBB

We used *in vitro* BBB model to determine whether the presence of ADMA affects adhesion or extravasation of primary human monocytes across the endothelial monolayer. ADMA enhanced the adhesion and migration of monocytes at a level comparable to inflammatory stimuli TNFα. Number of monocytes adhered within 40 min was 19,265 ± 1,035 (untreated), 30,411 ± 2,277 (TNFα treated), and 31,710 ± 1984 (ADMA treated) (Figure 2A; p < 0.001 with RM one-way ANOVA followed by Tukey’s *post hoc* test). The number of monocytes extravasated during 2 h via untreated hBMVEC monolayer was 3,103 ± 402; TNFa-treated 3,621 ± 434; ADMA-treated 4,908 ± 438 (Figure 2B; p = 0.015 with RM one-way ANOVA followed by Fisher’s LSD *post hoc* test).

### ADMA increases surface expression of VCAM-1 in hBMVEC

As we found that ADMA compromises barrier properties of hBMVEC and increases monocytes adhesion to and extravasation across the ADMA-activated hBMVEC, we seek to understand if ADMA could affect expression of cell adhesion molecules in hBMVEC. For this we used FACS to assess the surface expression of vascular cell adhesion protein-1 (VCAM-1), intracellular adhesion molecule-1 (ICAM-1), platelet endothelial cell adhesion molecule (PECAM-1) and activated leukocyte cell adhesion molecule (ALCAM-1). We used TNF-α as a positive control for endothelial activation. We found that 24 h treatment with ADMA elicited a significant increase in VCAM-1 surface expression level (Baseline (control) mean fluorescent intensity (MFI) was 2,747 ± 452.4 versus 5,048 ± 888.7 in ADMA treated group; p< 0.05 with RM followed by Tukey’s *post hoc* test; Figure 2C) and had no effect on ICAM-1 (MFI in control was 3,233 ± 298.2 versus 4,164 ± 385 in ADMA treated group; p> 0.05 with RM ANOVA followed by Tukey’s *post hoc* test; Figure 2D), PECAM-1 (MFI in control was 1,343 ± 59.4 versus 1,519 ± 77.7 in ADMA treated group; p> 0.05 with RM ANOVA followed by Tukey’s *post hoc* test; Figure 2E) and ALCAM-1 (MFI in control was 13,553 ± 674.5 versus 13,609 ± 621.9 in ADMA treated group; p> 0.05 with RM ANOVA followed by Tukey’s *post hoc* test; Figure 2F). TNF-α, at the same time was able to significantly increase surface expression levels for a broad range of adhesion molecules including VCAM-1 (MFI was 2,747 ± 452.4 in control versus 7,142 ± 927.5 in TNF-α treated group; p< 0.005 with RM ANOVA, followed by Tukey’s *post hoc* test; Figure 2C), ICAM-1 (MFI was 3,233 ± 298.2 in control versus 8,172 ± 1807 in TNF-α treated group; p< 0.005 with RM ANOVA, followed by Tukey’s *post hoc* test; Figure 2D), PECAM-1 (MFI was 1,343 ± 59.4 in control versus 1864 ± 185.6 in TNF-α treated group; p< 0.005 with RM ANOVA, followed by Tukey’s *post hoc* test; Figure 2E) and had no effect on ALCAM-1 (MFI in control was 13,553 ± 674.5 versus 13,713 ± 626.8 in TNF-α treated group; p> 0.05 with RM ANOVA followed by Tukey’s *post hoc* test; Figure 2F). Our results indicate that ADMA affects surface expression of adhesion molecules in a specific manner selectively increasing VCAM-1 in hBMVEC. (Figure 2C).

### ADMA triggers secretion of RANTES and VEGF-A from hBMVEC

To examine whether ADMA induces release of another markers of endothelial activation by hBMVEC, confluent cell monolayers were treated with 10 μM ADMA for 24 h. Cell culture supernatants were then analyzed by ELISA. Interestingly, the expression of the RANTES, VEGF-A, but not MCP-1 was significantly increased in ADMA-treated cells (Figures 3A, B). RANTES secretion by untreated cells was 9.775 ± 3.577 vs. 19.490 ± 4.219 by ADMA-treated cells, p < 0.001 with paired t-test. MCP-1 expression by hBMVEC was not changed by ADMA (1,367 ± 27.48 and 1,358 ± 22.44 pg/m; p > 0.5 with paired t-test). Angiogenic factor VEGF-A secretion was also enhanced by ADMA (Figure 3C; 1,031 ± 65,17 vs. 1,561 ± 65.68; p< 0.001 with paired t-test).

### ADMA triggers production of inflammatory cytokines in hBMVEC

**FIGURE 4:**
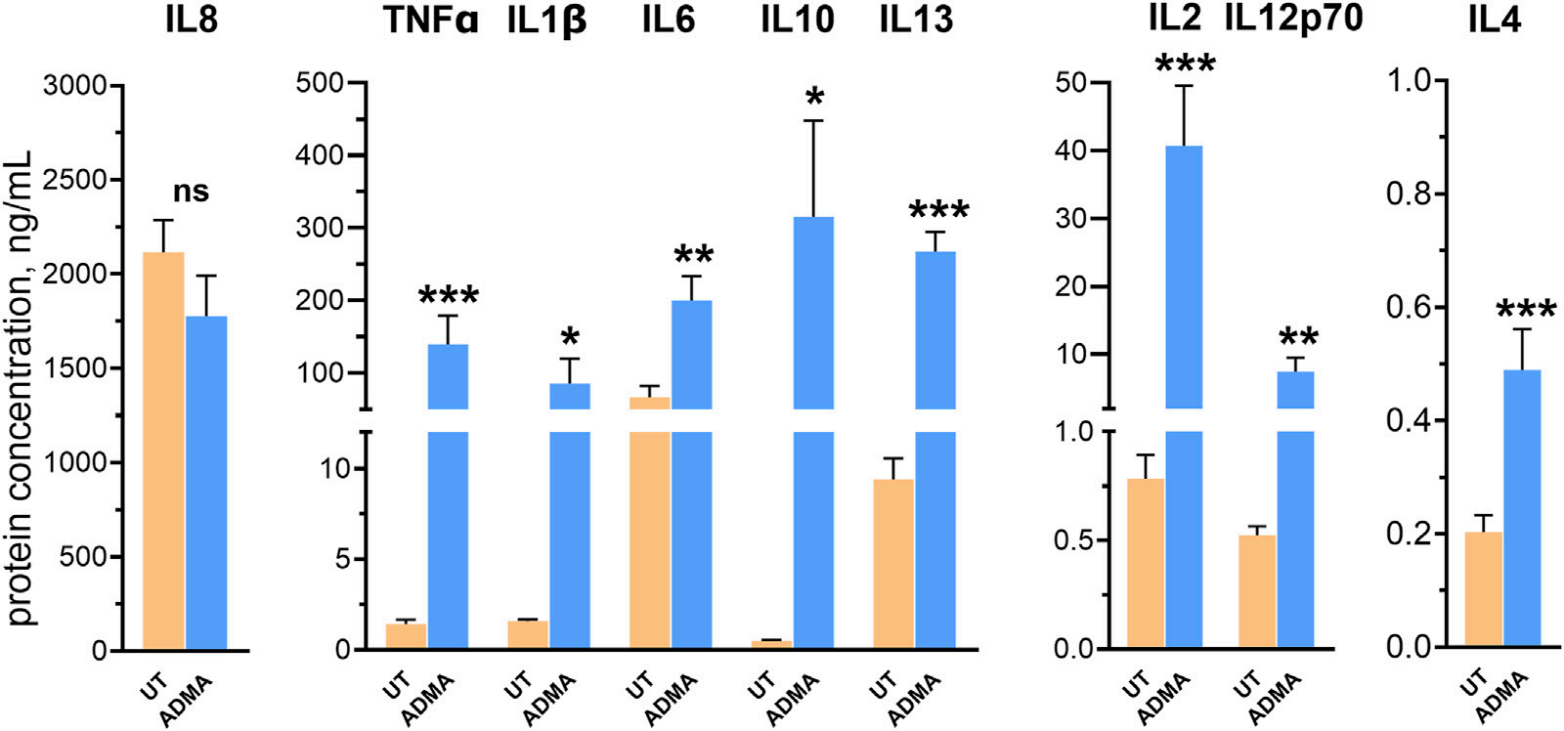
ADMA induces inflammatory activation of hBMVEC. hBMVEC were treated with 10 µM ADMA for 24 h and levels of cytokines were analyzed by V-PLEX assay. Levels of all analytes but IL-8 were significantly upregulated by the treatment. Data shown as the mean concentration ±SEM expressed in ng/mL. Experiments were performed using primary cells from four different donors (n = 8).

To examine whether ADMA induces release of pro- and anti-inflammatory cytokines by hBMVEC, confluent cell monolayers were treated with 10 μM ADMA for 24 h. Cell culture supernatants were then analyzed using the V-PLEX Proinflammatory Panels 1 Human kit (MesoScale Diagnostics), which includes interleukin IL-1β, IL-2, IL-4, IL-6, IL-8, IL-10, IL-12p70, IL-13, and TNFα. Out of nine analytes, the expression of eight cytokines was significantly increased in ADMA-treated cells (Figure 4). Concentrations of cytokines secreted by untreated cells vs. cells treated with ADMA (expressed in means ± SEM, ng/mL) were as follows: TNFα (1.423 ± 0.234 vs. 139.3 ± 39.32, p = 0.01 with paired t-test), IL-1β (1.574 ± 0.107 vs. 85.41 ± 34.07, p = 0.044 with paired t-test), IL-2 (0.784 ± 0.109 vs. 40.73 ± 8.850, p = 0.0226 with paired t-test), IL-4 (0.203 ± 0.03 vs. 0.489 ± 0.073, p = 0.0107 with paired t-test), IL-6 (66.49 ± 16.07 vs. 200.1 ± 33.43, p = 0.003 with paired t-test), IL-10 (0.4936 ± 0.0438 vs. 315.1 ± 133.1, p = 0.008 with Wilcoxon paired test), IL-12p70 (0.523 ± 0.0426 vs. 7.435 ± 2.093, p = 0.0127 with paired t-test) and IL-13 (9.404 ± 1.155 vs. 267.3 ± 27.06, p < 0.0001). It is only IL-8 for which there was no statistical significance when compared to the untreated control (2,116 ± 171.5 vs. 1776 ± 216.6, p = 0.124 with paired t-test). These results indicate that treatment with ADMA results in non-specific increase in both pro- and anti-inflammatory cytokines in hBMVEC.

## Discussion

Normally, intracellular concentration of ADMA in endothelial cells varies within 5–14 μM range, and ADMA concentration in the brain tissue is also reported to be about 5 µM (Böger et al., 2000; Cardounel and Zweier, 2002; Teerlink et al., 2009; Milewski et al., 2017). Here, we studied whether exogenously added ADMA can affect physical barrier properties of the endothelial monolayer. We found that incubation with ADMA for 36 h significantly decreased electrical resistance of hBMVEC monolayer, indicating a reduction in barrier properties. The effect of ADMA was developed in a time-dependent manner as 1, 10 and 100 µM of ADMA were able to reduce transendothelial resistance to the same extend but with a different efficacy over the time course of 36-h incubation. Previous study of Watson and colleagues with cerebral microvascular endothelial cell line hCMEC/D3 showed that ADMA is transported into cells by means of cationic acids transport system y+, presumably CAT-1, in a time-dependent manner (Watson et al., 2016). We suggest that difference in the time course for ADMA action on transendothelial resistance observed in our study can be associated with a concentration-dependent transport of ADMA into hBMVEC. We decided to further conduct all our experiments with 10 µM of ADMA as 1 µM of ADMA showed high variability in response and required more time to reach the same level of efficiency as 10 μM, while others show that 100 µM of ADMA can exert cytotoxic effect on hBMVEC and here we found that 10 µM does not affect viability of hBMVEC (Li et al., 2016).

Further we tested if ADMA could affect paracellular permeability in hBMVEC. We found that 10 µM of ADMA significantly increased permeability of hBMVEC within 3 h of incubation. Interestingly, previous studies found that treatment of hCMEC/D3, hBMVEC, and human umbilical vein endothelium cells (HUVECs) with ADMA for 24 h elicited increased permeability at high concentrations of ADMA (50–100 µM range or higher) (Wang et al., 2011; Li et al., 2016; Watson et al., 2016). The difference in our and previously published results can be explained by the fact that we used 4 kDa FITC-dextrans, while others were using 40 kDa FITC-dextran, which reflects the changes in transcellular rather than paracellular permeability route (Wang et al., 2011; Watson et al., 2016). Even though our and other studies using brain microvasculature endothelial cell lines showed that ADMA can increase BBB permeability the other studies investigating ADMA action in animal models of neurodegeneration and utilizing Evans Blue showed that chronic intraperitoneal injections with ADMA did not affect BBB permeability, and memory function in wild type control animals (Wang et al., 2011; Li et al., 2016; Watson et al., 2016; Choi et al., 2020; Singh et al., 2021). We suggest that this difference may be explained by *in vivo* ADMA delivery strategy as in that aforementioned *in vivo* studies ADMA was injected intraperitonially on a daily basis while other studies utilizing osmotic pumps for continuous intraperitoneal perfusion of ADMA showed significant decrease in locomotor activity, deficit in spatial memory and decrease in Claudin-1, Occludin and ZO-1 levels indicating disruption in BBB integrity in a brain tissue of healthy animals (Kielstein et al., 2015; Sheen et al., 2019; Choi et al., 2020; Hsu et al., 2021; 2023; Singh et al., 2021). These studies, though, did not aim to assess the BBB permeability directly. Thus, it is still an open question if ADMA can affect BBB permeability directly under *in vivo* conditions. Another important notice is that Evans Blue is a negatively charged molecule (neutral in a form of a salt) and we show that treatment with ADMA was able to increase hBMVEC monolayer permeability only for positively charged tracer with no effect for both negative and neutral tracers. Overall, the differences in ADMA-mediated BBB permeability for *in vitro* versus *in vivo* experiments still require further investigation.

Next, we tested if ADMA could affect immune-endothelial interactions at the BBB interface. Previously it was shown that ADMA increased ROS production and activation of transcription factor NF-κB in human cultured endothelial cell line (ECV 304) leading to increased adhesiveness of human leukemia monocytic cell line THP-1 (Böger et al., 2000), and that treatment with ADMA stimulated adhesion and migration of monocyte-derived dendritic cells (DC) via human dermal microvascular endothelial cell (HMVEC) monolayer (Weis et al., 2002). We are the first to show, that ADMA also caused activation of primary human brain endothelial cells and potentiate both adhesion and migration of primary human monocytes in the human *in vitro* BBB model providing cellular mechanisms underlaying ADMA-driven neuroimmune and neuroinflammatory responses.

Decreased electrical resistance of the endothelial barrier along with increased paracellular permeability, enhanced adhesion and extravasation of peripheral immune cells, all are strong indicators of ADMA-mediated BBB dysfunction. Therefore, we seek to determine what markers of endothelial activation are involved in ADMA-induced damage to the BBB. Several studies with HUVEC, human pulmonary artery endothelial cells (HPAEC), and ECV 304 reported that ADMA elicits activation of transcriptional factor NF-kB resulting in elevated expression of cell adhesion molecules (ICAM-1 and VCAM-1), chemokines (MCP-1 and RANTES), and cytokines TNF-α and IL-6 (Böger et al., 2000; Jiang et al., 2007; Zhang et al., 2011; Guo et al., 2013; Pekarova et al., 2015; Koudelka et al., 2016). Here, we are the first to report that ADMA specifically increases levels of VCAM-1, RANTES (CCL5) and VEGF-A in primary hBMVEC, while ICAM-1, PECAM-1, ALCAM-1, and MCP-1 (CCL2) remain unchanged. Interestingly, our immune-endothelial interaction results show that ADMA increases monocytes adhesion and extravasation to the levels comparable to TNF-α positive control, while not eliciting the same response in terms of the CAMS. One possible explanation could be that ADMA increases production of other molecules involved in monocytes adhesion and migration as chemokine RANTES (CCL5) and angiogenic factor VEGF-A that were elevated upon treatment with ADMA and are also known to increase monocytes adhesion and extravasation, however exact mechanisms requires further studies (Heil et al., 2000; von Hundelshausen et al., 2001; 2005; Keophiphath et al., 2010; Yang et al., 2014; Bus et al., 2018).

Next, we tested if ADMA can also modulate the neuroinflammatory profile of the BBB. We found that 24 h exposure to ADMA was sufficient to induce secretion of broad range of cytokines including pro-inflammatory (IL-1β, IL-2, TNF-a, IL-6), anti-inflammatory (IL-4, IL-10, IL-13) and cytokines involved in immune cells activation and adhesion (IL-4 and IL-2) (Carbone and Failla, 2021). Recently it was found that continuous infusion of ADMA decreases proteins responsible for BBB integrity and increases cytokine levels as IL-1α and IL-6 in hippocampus of young rats (Hsu et al., 2023). Our results indicate that increased brain inflammation observed in that study might be attributed to brain microvasculature endothelial cells activation associated with inflammatory response and infiltration of peripheral immune cells across BBB. In addition, overproduction of cytokines and activation of immune cells that we found in ADMA-treated hBMVECs allows to suggest ADMA as potential cause or comorbid factor for cytokine storm. Recently it was found that elevated ADMA serum concentration can predict in-hospital mortality of COVID-19 patients (Hannemann et al., 2021). Based on our results we can hypothesize that ADMA could be a possible comorbid factor causing or facilitating cytokine storm in SARS-CoV2 pathogenesis, yet this hypothesis requires further investigation. Overall, elevation in ADMA level accompanies vascular pathologies in cardiovascular and cerebrovascular disorders, atherosclerosis, neurodegeneration, as well as viral infections making asymmetric dimethylarginine an universal leading and/or comorbid factor for many pathological processes related to the disruption of endothelial function (Leong et al., 2008; Kurz et al., 2009; Sibal et al., 2010; Dorszewska, 2014; Dowsett et al., 2020; Bima et al., 2024).

It would be critical to stress upon some limitations of our study as in our *in vitro* BBB model we used only human brain microvasculature endothelial cells. It was found that ADMA can be accumulated and released by astrocytes and that ADMA can impair pericyte function in blood-retinal barrier (Milewski et al., 2017; Huang et al., 2019). Thus, additional studies with more complex cell models of human BBB and neurovascular unit can further straighten and expand our results providing new aspects for ADMA-mediated dysregulation of human BBB function.

To summarize, our results corroborate with previously published reports and provides new aspects of ADMA-induced effects in human brain, showing that at clinically relevant concentrations ADMA negatively modulates physical barrier properties of the BBB, elicits neuroinflammatory responses in cerebral endothelium, and stimulates infiltration of peripheral immune cells.

## Data Availability

The raw data supporting the conclusions of this article will be made available by the authors upon the request.

## Supplementary material

SUPPLEMENTARY FIGURE 1 ADMA does not affect the viability of hBMVEC. **(A)** Treatment (24 h) and **(B)** Treatment (72 h) with 10 µM ADMA did not cause significant toxicity or cell death compared with that in untreated controls. 0.1% Saponin (sap) was used as a positive control. Data is presented as the mean number of live cells ±SEM. Data is presented as the mean number of live cells ±SEM and mean number of dead cells ± SEM. Experiments were independently performed at least three times and primary cells from 5 different donors were used (n = 12).

SUPPLEMENTARY FIGURE 2 Time-response of 1, 10 and 100 µM ADMA or 100 ng/mL TNFα action on barrier electrical resistance of hBMVEC monolayer at 1 h **(A)**, 3 h **(B)**, 9 h **(C)**, 18 h **(D)**, 24 h **(E)** and 36 h **(F)** time points. Treatments were initiated at 0 timepoint. Each data point is represented as the percentage of the mean value ±SEM. Experiments were independently performed three times, primary cells from four different donors were used (n = 14).
