# Supplementary figures and images for: Asymmetric dimethylarginine induces maladaptive function of the blood-brain barrier
Source: Front Cell Dev Biol. 2024 Oct 9;12:1476386. doi: 10.3389/fcell.2024.1476386 (PMC11496185; doi:10.3389/fcell.2024.1476386)

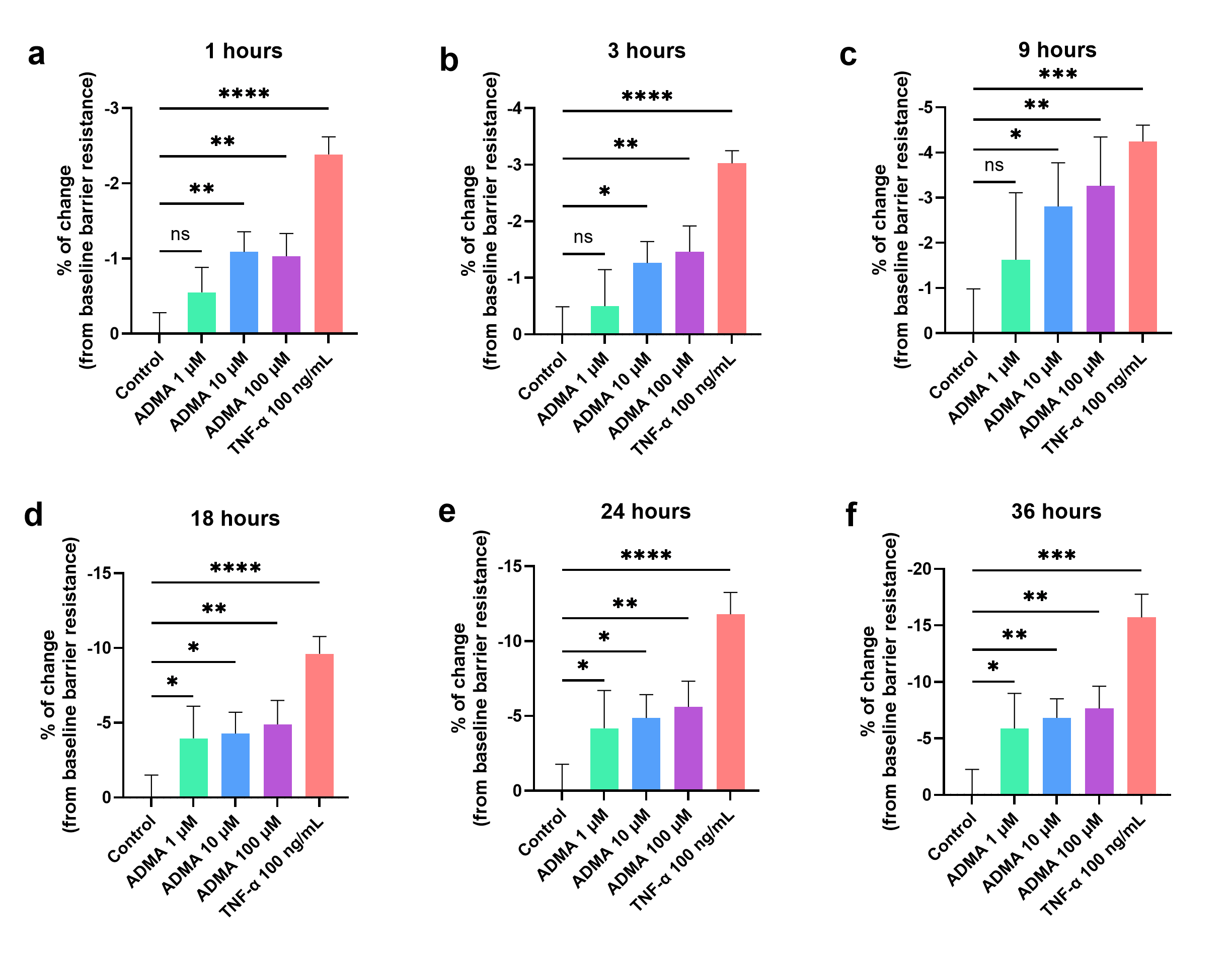

Supplement: Supplementary file 1 [file Image2.TIF]

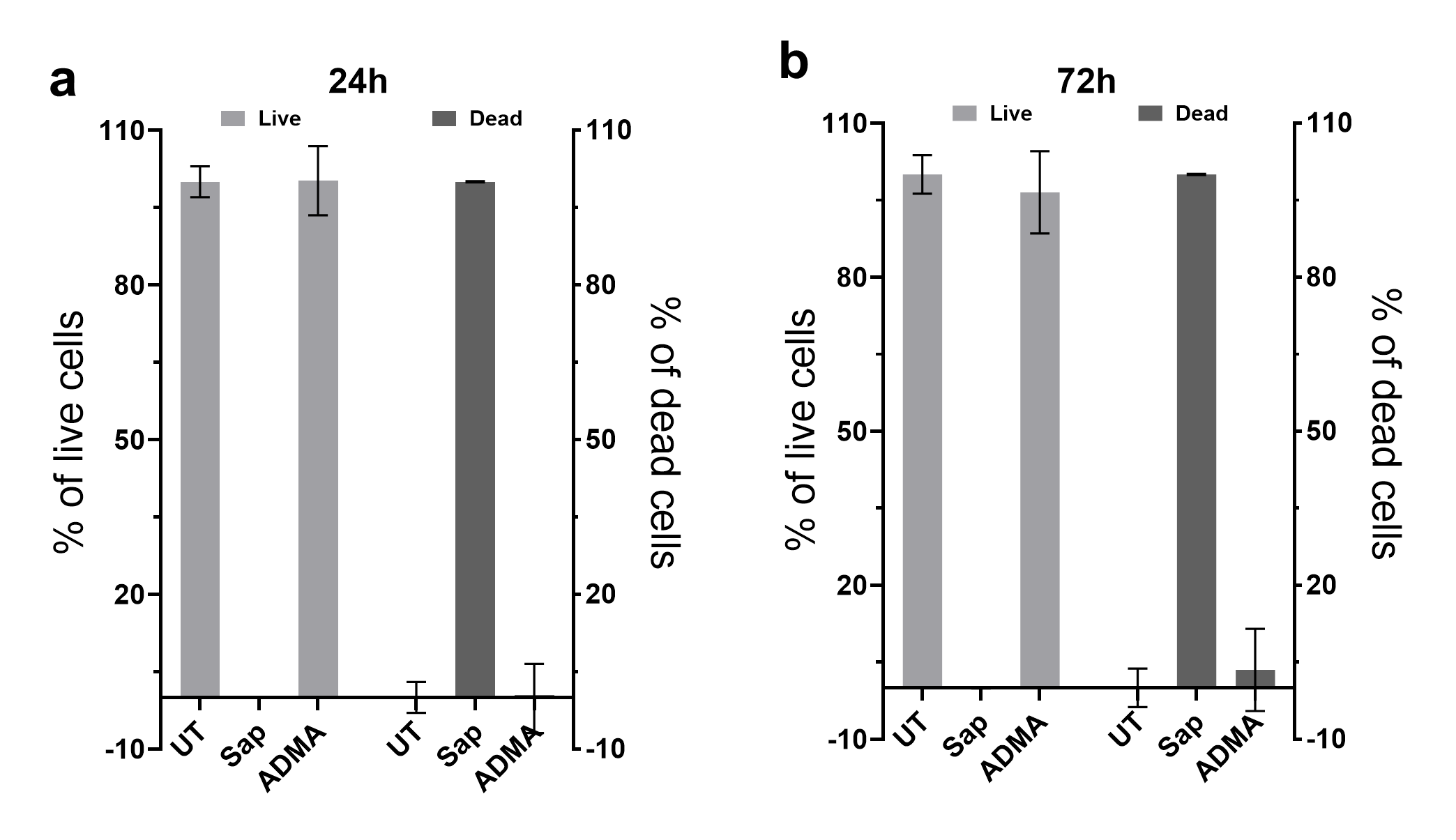

Supplement: Supplementary file 2 [file Image1.TIF]
